# Is There a Need for Protein Ingestion During Exercise?

**DOI:** 10.1007/s40279-014-0156-z

**Published:** 2014-05-03

**Authors:** Luc J. C. van Loon

**Affiliations:** Department of Human Movement Sciences, NUTRIM School for Nutrition, Toxicology and Metabolism, Maastricht University Medical Centre+, PO Box 616, 6200 MD Maastricht, The Netherlands

## Abstract

Dietary protein ingestion following exercise increases muscle protein synthesis rates, stimulates net muscle protein accretion, and facilitates the skeletal muscle adaptive response to prolonged exercise training. Furthermore, recent studies show that protein ingestion before and during exercise also increases muscle protein synthesis rates during resistance- and endurance-type exercise. Therefore, protein ingestion before and during prolonged exercise may represent an effective dietary strategy to enhance the skeletal muscle adaptive response to each exercise session by extending the window of opportunity during which the muscle protein synthetic response is facilitated. Protein ingestion during exercise has also been suggested to improve performance capacity acutely. However, recent studies investigating the impact of protein ingestion during exercise on time trial performance, as opposed to time to exhaustion, do not report ergogenic benefits of protein ingestion. Therefore, it is concluded that protein ingestion with carbohydrate during exercise does not further improve exercise performance when compared with the ingestion of ample amounts of carbohydrate only.

## Introduction

Skeletal muscle tissue has an enormous capacity to adapt structurally to changes in muscle use or disuse. This allows humans to adapt to more prolonged exercise training, thereby increasing performance capacity. This plasticity of skeletal muscle becomes most evident when the large differences in the structural adaptation to prolonged resistance are compared with endurance-type exercise training, with each having its distinct phenotypic outcome. Simply compare the physique of a professional bodybuilder with that of a triathlete. This muscle plasticity is facilitated by the fact that skeletal muscle tissue has a turnover rate of 1–2 % per day, with muscle protein synthesis rates ranging between 0.04 and 0.14 % per hour. The rate of skeletal muscle protein synthesis is regulated by two main anabolic stimuli: food intake and physical activity.

Food intake, or rather protein ingestion, directly elevates muscle protein synthesis rates [1–3]. The dietary protein-derived amino acids act as key signaling proteins activating anabolic pathways in skeletal muscle tissue, and provide precursors for de novo muscle protein synthesis. Ingestion of a meal-like amount of dietary protein (15–20 g) elevates muscle protein synthesis rates for 2–5 h following meal ingestion [2], resulting in net muscle protein accretion [2]. The other main anabolic stimulus is physical activity. Physical activity (or exercise) directly stimulates skeletal muscle protein synthesis [4–6], an effect that has been shown to persist for up to 48 h after the cessation of exercise [6]. Of course, different types of exercise will stimulate the synthesis of different sets of proteins. Whereas resistance-type exercise strongly stimulates the synthesis of myofibrillar proteins, endurance-type exercise will have a greater impact on stimulating the synthesis of mitochondrial proteins [2], thereby allowing exercise-specific skeletal muscle adaptation.

Athletes, coaches, and scientists are well aware of the impact of both exercise and nutrition in facilitating the skeletal muscle adaptive response to exercise training. Consequently, much work is being done to define dietary strategies that facilitate the adaptive response to prolonged exercise training and improve exercise training efficiency. This paper discusses the potential benefits of dietary protein ingestion during exercise as a means to support the adaptive response to exercise training. Furthermore, there are also suggestions that amino acid or protein ingestion during exercise can directly augment exercise performance. Therefore, this paper also discusses the proposed direct ergogenic properties of amino acid or protein ingestion during exercise.

## Exercise and Nutrition

A single bout of exercise increases skeletal muscle protein synthesis and, to a lesser extent, muscle protein breakdown rates, thereby improving muscle protein balance [6]. Although exercise improves muscle protein balance, net protein balance will remain negative in the absence of food intake. In other words, nutrition is required to allow proper muscle reconditioning and is a prerequisite for muscle hypertrophy to occur. As such, it is not surprising that a strong synergy exists between exercise and nutrition.

When protein is ingested following a single bout of exercise, muscle protein synthesis rates are increased to a much higher level and for a more prolonged period of time than in a normal postprandial muscle protein synthetic response [2]. Moreover, recent work from the author’s laboratory has shown that when exercise is performed before the ingestion of a meal-like amount of dietary protein, more of the ingested protein is used for de novo muscle protein accretion [3]. Consequently, the metabolic fate of ingested protein may largely depend on the amount of physical activity that is performed before food consumption. The stimulating properties of exercise on the postprandial muscle protein synthetic response are long lived and persist over an extended period of time, lasting as much as 24 h after performing a single bout of exercise [7]. The latter is in line with previous work showing that protein supplementation represents an effective dietary strategy to augment further the skeletal muscle adaptive response to more prolonged resistance-type exercise training, resulting in greater gains in skeletal muscle mass and strength [8–19]. However, many other studies have also failed to detect a surplus benefit of dietary protein supplementation on the skeletal muscle adaptive response to more prolonged resistance-type exercise training [20–38].

A meta-analysis was recently performed in an attempt to explain these apparently discrepant findings on the proposed efficacy of dietary protein supplementation as a means to increase the gains in skeletal muscle mass and strength during prolonged resistance-type exercise training [39]. The outcome of this meta-analysis confirmed the surplus benefits of dietary protein supplementation, but also revealed a large variance in the observed impact of nutritional co-intervention on the skeletal muscle adaptive response to a prolonged resistance-type exercise training program. Obviously, there are still considerable challenges ahead to define the most effective nutritional intervention strategies that can further optimize exercise-induced skeletal muscle reconditioning.

Many research groups are presently studying the various individual factors that may augment the acute post-exercise muscle protein synthetic response. Various studies have previously assessed the impact of the amount [40] and type [41–43] of dietary protein that is ingested following an exercise bout on subsequent post-exercise muscle protein synthesis rates. Others have assessed the impact of co-ingesting specific free amino acids [44], other macronutrients [45, 46], and/or specific nutritional compounds [47] that may further enhance post-exercise muscle protein synthesis. It is beyond the scope of this paper to discuss all dietary factors that may augment post-exercise muscle protein synthesis. Therefore, it focuses on a single parameter that is likely to be of key importance in driving the muscle protein synthetic response to exercise: the timing of protein provision.

## Timing of Protein Ingestion

Besides the amount and type of protein ingested during post-exercise recovery, the timing of protein ingestion has been identified as another key factor modulating post-exercise muscle protein anabolism. Levenhagen et al. [48] were one of the first groups to report a more positive net protein balance after consuming a protein (containing) supplement immediately after exercise when compared with the provision of the same supplement 3 h into post-exercise recovery. As a consequence, it is now generally advised to ingest 20–25 g of a high-quality dietary protein (dairy or meat) immediately after the cessation of exercise as a means to optimize post-exercise reconditioning [40].

More recent work suggests that protein may even be consumed before and/or during exercise to stimulate post-exercise muscle protein accretion further [49–52]. Tipton et al. [52] reported that the ingestion of a mixture of 6 g of essential amino acids and 35 g of sucrose before exercise was more effective for the stimulation of post-exercise muscle protein synthesis than ingesting the same mixture immediately after exercise. The authors hypothesized that the greater stimulation of muscle protein synthesis may be attributed to the combination of increased amino acid levels at a time when blood flow is increased during exercise, thereby offering a greater stimulation of muscle protein synthesis by increasing amino acid delivery to the muscle. In a subsequent study, the same research group failed to confirm these findings when examining the impact of 20 g of whey protein ingested before as opposed to 1 h after resistance-type exercise on muscle protein balance measured over a 4- to 5-h recovery period [53]. It seems likely that the longer recovery period in the second study at least partly compensated for any early benefits of protein provision before exercise as protein synthesis rates tend to reach peak values within 2–3 h after exercise. Protein ingestion before or during exercise allows post-exercise muscle protein synthesis rates to be elevated more rapidly due to the greater amino acid availability to the muscle during the early stages of post-exercise recovery.

**Fig. 1:**
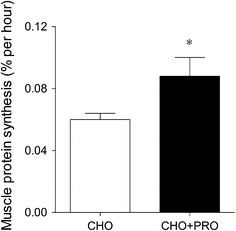
Dietary protein ingestion before and during resistance-type exercise stimulates muscle protein synthesis during exercise. Fractional synthesis rate of mixed muscle protein during exercise following carbohydrate (CHO) or carbohydrate plus protein (CHO + PRO) ingestion. Values represent means ± standard error of the mean. * Significantly different from carbohydrate. Reproduced with modification from Beelen et al. [49]

The ingestion of protein before or during exercise could be of even more benefit during the early stages of recovery from more intense exercise bouts. Exhaustive exercise is generally accompanied by a redistribution of blood flow to skeletal muscle tissue, resulting in hypoperfusion of the gut [54]. The exercise-induced hypoperfusion of the gut induces intestinal damage and impairs dietary protein digestion and absorption kinetics during early post-exercise recovery [55]. Dietary protein ingestion before and/or during exercise may provide a more effective feeding strategy to improve amino acid availability during early post-exercise recovery. The author’s laboratory performed a series of studies to assess the impact of protein provision before and during exercise on muscle protein synthesis rates measured during exercise conditions [49–51]. In a first study, recreational athletes ingested carbohydrate-containing drinks (0.15 g/kg body mass/h) with or without additional protein (0.15 g/kg body mass/h) before and during 2 h of resistance-type exercise. Using contemporary stable isotope methodology, it was shown that protein co-ingestion before and during resistance-type exercise substantially increased muscle protein synthesis rates during exercise conditions (Fig. 1) [49]. The observation that muscle protein synthesis rates can be elevated during exercise could be of particular relevance as it may extend the window of opportunity during which the muscle protein synthetic response to exercise can be facilitated.

It could be speculated that the observed impact of protein ingestion with carbohydrate on mixed-muscle protein synthesis during exercise is merely restricted to intermittent, resistance-type exercise activities [49, 56]. Adenosine monophosphate-activated protein kinase may not be continually activated throughout intermittent, resistance-type exercise when exercise is performed in the fed state. The latter may prevent its proposed inhibitory effect on muscle protein synthesis, allowing muscle protein accretion during rest periods in between sets of exercise.

It has been debated whether dietary protein administration before and/or during exercise may stimulate muscle protein synthesis during continuous endurance-type exercise activities. Previous work has clearly shown that protein co-ingestion with carbohydrate during more prolonged endurance-type exercise improves whole-body protein balance [51]. Moreover, whereas whole-body protein balance remained negative when only carbohydrates were ingested, dietary protein co-ingestion was shown to improve whole-body protein balance by increasing protein synthesis as well as decreasing protein breakdown, resulting in a positive protein balance during 5 h of prolonged endurance-type exercise. As measurements on a whole-body level do not necessarily reflect skeletal muscle tissue, a follow-up study was performed to assess muscle protein synthesis rates during endurance-type exercise while ingesting carbohydrate or carbohydrate plus protein. Interestingly, that study showed that muscle protein synthesis rates were higher during exercise than were pre-exercise post-absorptive protein synthesis rates [50]. When evaluating myocellular signaling, it became apparent that activation/phosphorylation of both adenosine monophosphate-activated protein kinase as well as mammalian target of rapamycin can occur in parallel in vivo in mixed muscle tissue. However, no significant differences were observed in muscle protein synthesis rates between the carbohydrate and carbohydrate plus protein trial, despite clear differences in whole-body protein balance.

Future studies are warranted to assess muscle protein synthesis rates during more prolonged exercise (3–5 h), as longer exercise trials will allow differences in fractional protein synthesis rates to become more apparent. Clearly, more work is also needed to address the relevance of the potential to stimulate muscle protein synthesis during resistance- and endurance-type exercise activities, thereby creating a longer time frame for muscle protein synthesis rates to be increased. This may be of particular relevance for the (ultra)endurance athletes who spend many hours exercising.

## Window of Opportunity

So what is the preferred timing of dietary protein supplementation when trying to optimize the skeletal muscle adaptive response to successive exercise sessions? Although this seems a simple question, the answer is rather complicated. Exercise increases muscle protein synthesis rates for several hours after a single bout of exercise. Protein ingestion further augments the post-exercise muscle protein synthetic response. As such, it is not surprising that protein supplementation during prolonged resistance-type exercise training generally leads to greater gains in skeletal muscle mass and/or strength.

It is generally advised to provide 20–25 g of a high-quality protein immediately after an exercise session to maximize the muscle protein synthetic response during acute post-exercise recovery [57]. However, the window of opportunity to allow muscle protein synthesis rates to be elevated is not limited to these few hours of acute post-exercise recovery. Muscle protein synthesis is already stimulated during exercise when protein is provided before and/or during exercise. The latter may extend the window of opportunity and accelerate skeletal muscle reconditioning. So it might be particularly wise to ingest some dietary protein before and during more prolonged (ultra)endurance-type exercise bouts (>3–5 h). The latter may prevent excess muscle protein breakdown and allow muscle protein synthesis to be elevated throughout the exercise sessions. Such a dietary strategy may facilitate muscle reconditioning and improve training efficiency. Nonetheless, this still provides a very simplistic idea of the role of nutrition and exercise training in skeletal muscle reconditioning. The skeletal muscle adaptive response to exercise is not limited to the exercise session itself and the subsequent hours of acute post-exercise recovery. It has been reported that basal muscle protein synthesis rates as well as the muscle protein synthetic response to food intake are increased up to 24 h after a resistance-type exercise session [7]. Such findings are interesting and imply that the window of opportunity to modulate the skeletal muscle adaptive response to exercise is much larger and may also depend on the overall type of training [58].

The window of opportunity probably also extends to overnight recovery during sleep. Because of obvious methodological issues, this has hardly been studied. The impact of exercise performed in the evening on muscle protein synthesis during subsequent overnight recovery was recently evaluated [59]. Although an increase in muscle protein synthesis was observed during the first few hours of acute post-exercise recovery, muscle protein synthesis rates remained unexpectedly low during overnight sleep. Whereas dietary protein ingestion after the cessation of exercise stimulates muscle protein synthesis during the acute stages of post-exercise recovery, these elevated synthesis rates do not seem to be maintained during subsequent overnight sleep.

Using various models, it has now been established that protein administration before sleep (by means of ingestion [60]) or during sleep (by means of nasogastric tube feeding [61]) is followed by normal dietary protein digestion and absorption, increasing plasma amino acid availability, and stimulating net muscle protein accretion throughout overnight sleep. Consequently, the night provides another interesting extension of the window of opportunity during which the adaptive process can be facilitated. It will be challenging to define whether there is an actual limited ‘window of opportunity’ for nutritional interventions to improve skeletal muscle reconditioning further. Clearly, it is too early to provide a definite answer on the impact of the distribution of dietary protein provided throughout the day (and night) to maximize the exercise training response.

## Protein as an Ergogenic Aid

**Fig. 2:**
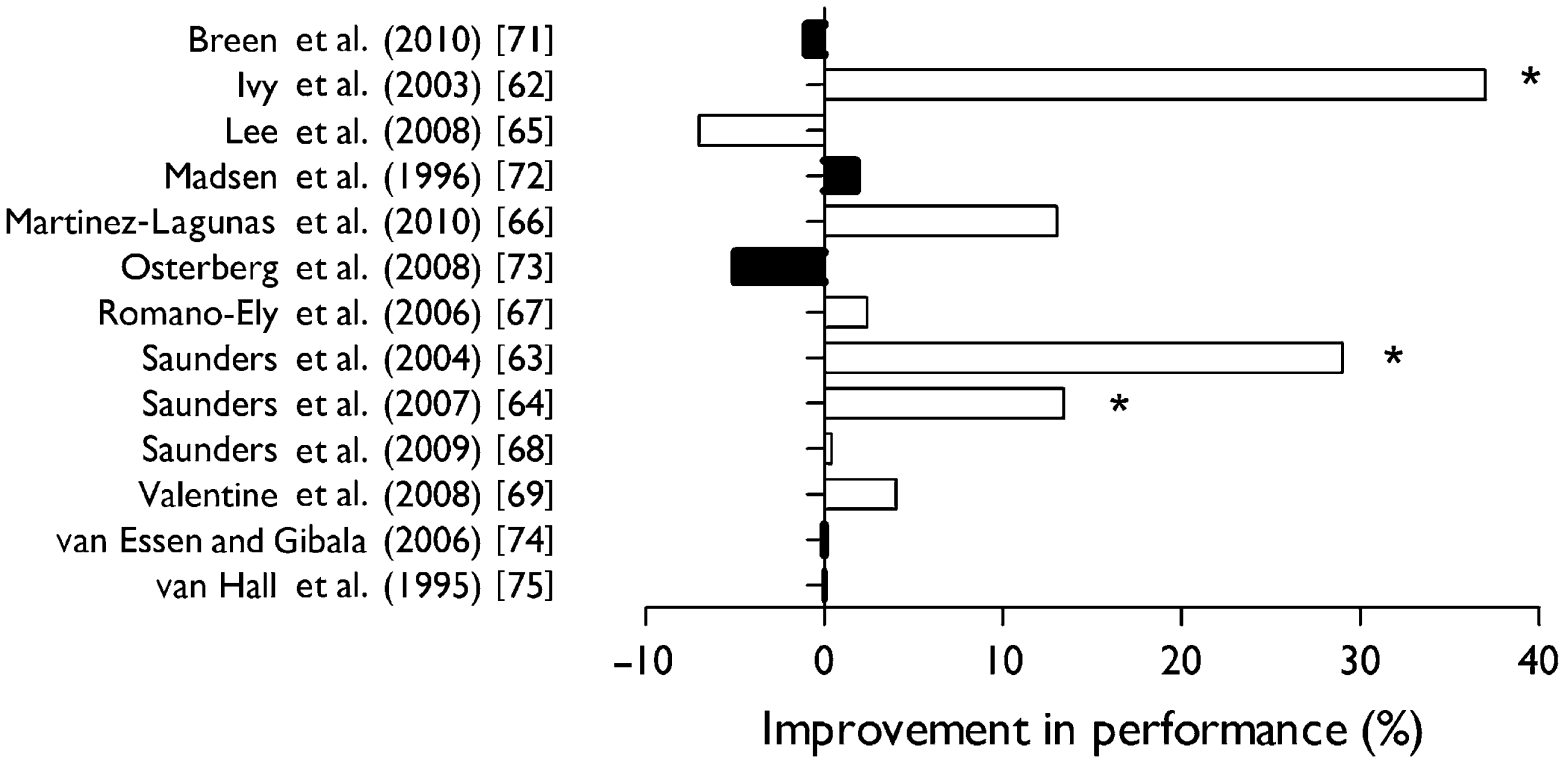
An overview of studies investigating the impact of dietary protein ingestion during endurance-type exercise on subsequent performance capacity. Performance capacity was assessed either as time to exhaustion (*open bars*) or as time trial performance (*filled bars*) in the various studies. * Significant improvement in performance reported following protein co-ingestion during exercise

Dietary protein ingestion during and/or immediately after each exercise bout facilitates muscle reconditioning and may help to improve training efficiency. However, over the past few years there have also been suggestions that protein ingestion during exercise may directly improve performance during competition. Ivy et al. [62] published the first paper in which they reported increased performance capacity in trained cyclists following the ingestion of carbohydrate plus protein during prolonged cycling. Nine cyclists were recruited and performed cycling exercise until exhaustion while ingesting drinks containing carbohydrate, carbohydrate plus protein, or flavored water. The authors reported that the ingestion of a carbohydrate solution with added protein enhanced endurance performance when compared with the ingestion of the carbohydrate solution only. However, the reason for this improvement in performance remained unclear. Since then, additional studies have been published [63, 64] reporting a significantly greater time to exhaustion following the ingestion of carbohydrate plus protein during more prolonged endurance-type exercise tasks (Fig. 2). More recent studies have been unable to confirm these findings [65–69].

Clearly, some studies have reported substantial (10–30 %) improvements in time to exhaustion, implying that protein co-ingestion during exercise represents an effective dietary strategy to improve performance capacity [62–64]. Besides the fact that there are no apparent mechanistic explanations for the observed impact of protein co-ingestion on time to exhaustion, it is unlikely that such large increases in time to exhaustion will translate to similar improvements in exercise performance in a more practical sports setting that simulates normal athletic competition.

When evaluating the impact of sports drinks on exercise performance it is generally preferred to assess time trial performance. Time trials have shown a greater validity than time to exhaustion trials because they provide a good physiological simulation of actual performance [70]. Consequently, several studies have investigated the proposed acute ergogenic benefits of carbohydrate plus protein ingestion on time trial performance (Fig. 2). However, none of these studies has detected any acute performance-enhancing effects of amino acid or protein ingestion during exercise [71–75]. In short, dietary protein ingestion with carbohydrate during exercise does not improve exercise performance above carbohydrate ingestion alone when ample carbohydrates are ingested. Therefore, there is no support to suggest that protein should be ingested with carbohydrate during competition to maximize exercise performance.

## Conclusion

Dietary protein ingestion after exercise increases post-exercise muscle protein synthesis rates, stimulates net muscle protein accretion, and facilitates the skeletal muscle adaptive response to prolonged exercise training. Recent studies have shown that protein ingestion before and during exercise stimulates muscle protein synthesis during exercise. Therefore, protein ingestion before and/or during prolonged exercise training sessions may inhibit muscle protein breakdown, stimulate muscle protein synthesis, and further augment the skeletal muscle adaptive response to exercise training. The ingestion of dietary protein with carbohydrate during exercise does not further enhance performance capacity when compared with the ingestion of ample amounts of carbohydrate only, but may be applied to improve exercise reconditioning.

## References

[1] Koopman R, Verdijk L, Manders RJ (2006). Co-ingestion of protein and leucine stimulates muscle protein synthesis rates to the same extent in young and elderly lean men. Am J Clin Nutr.

[2] Moore DR, Tang JE, Burd NA (2009). Differential stimulation of myofibrillar and sarcoplasmic protein synthesis with protein ingestion at rest and after resistance exercise. J Physiol.

[3] Pennings B, Koopman R, Beelen M (2010). Exercising before protein intake allows for greater use of dietary protein-derived amino acids for de novo muscle protein synthesis in both young and elderly men. Am J Clin Nutr.

[4] Biolo G, Maggi SP, Williams BD (1995). Increased rates of muscle protein turnover and amino acid transport after resistance exercise in humans. Am J Physiol.

[5] Chesley A, MacDougall JD, Tarnopolsky MA (1992). Changes in human muscle protein synthesis after resistance exercise. J Appl Physiol.

[6] Phillips SM, Tipton KD, Aarsland A (1997). Mixed muscle protein synthesis and breakdown after resistance exercise in humans. Am J Physiol.

[7] Burd NA, West DW, Moore DR (2011). Enhanced amino acid sensitivity of myofibrillar protein synthesis persists for up to 24 h after resistance exercise in young men. J Nutr.

[8] Andersen LL, Tufekovic G, Zebis MK (2005). The effect of resistance training combined with timed ingestion of protein on muscle fiber size and muscle strength. Metabolism.

[9] Bird SP, Tarpenning KM, Marino FE (2006). Independent and combined effects of liquid carbohydrate/essential amino acid ingestion on hormonal and muscular adaptations following resistance training in untrained men. Eur J Appl Physiol.

[10] Burke DG, Chilibeck PD, Davidson KS (2001). The effect of whey protein supplementation with and without creatine monohydrate combined with resistance training on lean tissue mass and muscle strength. Int J Sport Nutr Exerc Metab.

[11] Candow DG, Burke NC, Smith-Palmer T (2006). Effect of whey and soy protein supplementation combined with resistance training in young adults. Int J Sport Nutr Exerc Metab.

[12] Coburn JW, Housh DJ, Housh TJ (2006). Effects of leucine and whey protein supplementation during eight weeks of unilateral resistance training. J Strength Cond Res.

[13] Cribb PJ, Williams AD, Hayes A (2007). A creatine–protein–carbohydrate supplement enhances responses to resistance training. Med Sci Sports Exerc.

[14] Hartman J, Tang JE, Wilkinson SB (2007). Consumption of fat-free fluid milk after resistance exercise promotes greater lean mass accretion than does consumption of soy or carbohydrate in young, novice, male weightlifters. Am J Clin Nutr.

[15] Josse AR, Tang JE, Tarnopolsky MA (2010). Body composition and strength changes in women with milk and resistance exercise. Med Sci Sports Exerc.

[16] Kerksick CM, Rasmussen CJ, Lancaster SL (2006). The effects of protein and amino acid supplementation on performance and training adaptations during ten weeks of resistance training. J Strength Cond Res.

[17] Vieillevoye S, Poortmans JR, Duchateau J (2010). Effects of a combined essential amino acids/carbohydrate supplementation on muscle mass, architecture and maximal strength following heavy-load training. Eur J Appl Physiol.

[18] Walker TB, Smith J, Herrera M (2010). The influence of 8 weeks of whey-protein and leucine supplementation on physical and cognitive performance. Int J Sport Nutr.

[19] Willoughby DS, Stout JR, Wilborn CD (2007). Effects of resistance training and protein plus amino acid supplementation on muscle anabolism, mass, and strength. Amino Acids.

[20] Antonio J, Sanders MS, Ehler LA (2000). Effects of exercise training and amino-acid supplementation on body composition and physical performance in untrained women. Nutrition.

[21] Ballard T, Specker B, Binkley T (2006). Effect of protein supplementation during a 6-month strength and conditioning program on areal and volumetric bone parameters. Bone.

[22] Beck T, Housh T, Johnson G (2007). Effects of a drink containing creatine, amino acids, and protein combined with ten weeks of resistance training on body composition, strength, and anaerobic performance. J Strength Cond Res.

[23] Bemben MG, Witten MS, Carter DL (2010). The effects of supplementation with creatine and protein on muscle strength following a traditional resistance training program in middle-aged and older men. J Nutr Health Aging.

[24] Campbell W, Crim M, Young V (1995). Effects of resistance training and dietary protein intake on protein metabolism in older adults. Am J Physiol.

[25] Chromiak JA, Smedley B, Carpenter W (2004). Effect of a 10-week strength training program and recovery drink on body composition, muscular strength and endurance, and anaerobic power and capacity. Nutrition.

[26] Hoffman JR, Ratamess NA, Kang J (2007). Effects of protein supplementation on muscular performance and resting hormonal changes in college football players. J Sports Sci Med.

[27] Hoffman JR, Ratamess NA, Tranchina CP (2009). Effect of protein-supplement timing on strength, power, and body-composition changes in resistance-trained men. Int J Sport Nutr Exerc Metab.

[28] Holm L, Olesen JL, Matsumoto K (2008). Protein-containing nutrient supplementation following strength training enhances the effect on muscle mass, strength, and bone formation in postmenopausal women. J Appl Physiol.

[29] Hulmi JJ, Kovanen V, Selanne H (2009). Acute and long-term effects of resistance exercise with or without protein ingestion on muscle hypertrophy and gene expression. Amino Acids.

[30] Hulmi JJ, Tannerstedt J, Selanne H (2009). Resistance exercise with whey protein ingestion affects mTOR signaling pathway and myostatin in men. J Appl Physiol.

[31] Iglay H, Apolzan J, Gerrard D (2009). Moderately increased protein intake predominately from egg sources does not influence whole body, regional, or muscle composition responses to resistance training in older people. J Nutr Health Aging.

[32] Kukuljan S, Nowson CA, Sanders K (2009). Effects of resistance exercise and fortified milk on skeletal muscle mass, muscle size, and functional performance in middle-aged and older men: an 18-mo randomized controlled trial. J Appl Physiol.

[33] Lemon PW, Tarnopolsky MA, MacDougall JD (1992). Protein requirements and muscle mass/strength changes during intensive training in novice bodybuilders. J Appl Physiol.

[34] Mielke M, Housh TJ, Malek MH (2009). The effects of whey protein and leucine supplementation on strength, muscular endurance, and body composition during resistance training. J Exerc Physiol.

[35] Rozenek R, Ward P, Long S (2002). Effects of high-calorie supplements on body composition and muscular strength following resistance training. J Sports Med Phys Fitness.

[36] Verdijk L, Jonkers R, Gleeson B (2009). Protein supplementation before and after exercise does not further augment skeletal muscle hypertrophy after resistance training in elderly men. Am J Clin Nutr.

[37] Walberg Rankin J, Goldman LP, Puglisi MJ (2004). Effect of post-exercise supplement consumption on adaptations to resistance training. J Am Coll Nutr.

[38] White K, Bauer S, Hartz K (2009). Changes in body composition with yogurt consumption during resistance training in women. Int J Sports Nutr Exerc Metab.

[39] Cermak NM, Res PT, de Groot LCPGM (2012). Protein supplementation augments the skeletal muscle adaptive response to resistance-type exercise training: a meta-analysis. Am J Clin Nutr.

[40] Moore DR, Robinson MJ, Fry JL (2009). Ingested protein dose response of muscle and albumin protein synthesis after resistance exercise in young men. Am J Clin Nutr.

[41] Tang JE, Moore DR, Kujbida GW (2009). Ingestion of whey hydrolysate, casein, or soy protein isolate: effects on mixed muscle protein synthesis at rest and following resistance exercise in young men. J Appl Physiol.

[42] Tipton KD, Elliott TA, Cree MG (2004). Ingestion of casein and whey proteins result in muscle anabolism after resistance exercise. Med Sci Sports Exerc.

[43] Wilkinson SB, Tarnopolsky MA, Macdonald MJ (2007). Consumption of fluid skim milk promotes greater muscle protein accretion after resistance exercise than does consumption of an isonitrogenous and isoenergetic soy-protein beverage. Am J Clin Nutr.

[44] Koopman R, Verdijk LB, Beelen M (2008). Co-ingestion of leucine with protein does not further augment post-exercise muscle protein synthesis rates in elderly men. Br J Nutr.

[45] Glynn EL, Fry CS, Drummond MJ (2011). Muscle protein breakdown has a minor role in the protein anabolic response to essential amino acid and carbohydrate intake following resistance exercise. Am J Physiol.

[46] Koopman R, Beelen M, Stellingwerff T (2007). Coingestion of carbohydrate with protein does not further augment postexercise muscle protein synthesis. Am J Physiol.

[47] Smith GI, Atherton P, Reeds DN (2011). Dietary omega-3 fatty acid supplementation increases the rate of muscle protein synthesis in older adults: a randomized controlled trial. Am J Clin Nutr.

[48] Levenhagen DK, Gresham JD, Carlson MG (2001). Postexercise nutrient intake timing in humans is critical to recovery of leg glucose and protein homeostasis. Am J Physiol.

[49] Beelen M, Koopman R, Gijsen AP (2008). Protein coingestion stimulates muscle protein synthesis during resistance-type exercise. Am J Physiol.

[50] Beelen M, Zorenc A, Pennings B (2011). Impact of protein coingestion on muscle protein synthesis during continuous endurance type exercise. Am J Physiol.

[51] Koopman R, Pannemans DL, Jeukendrup AE (2004). Combined ingestion of protein and carbohydrate improves protein balance during ultra-endurance exercise. Am J Physiol.

[52] Tipton KD, Rasmussen BB, Miller SL (2001). Timing of amino acid–carbohydrate ingestion alters anabolic response of muscle to resistance exercise. Am J Physiol.

[53] Tipton KD, Elliott TA, Cree MG (2007). *S*timulation of net muscle protein synthesis by whey protein ingestion before and after exercise. Am J Physiol.

[54] van Wijck K, Lenaerts K, Grootjans J (2012). Physiology and pathophysiology of splanchnic hypoperfusion and intestinal injury during exercise: strategies for evaluation and prevention. Am J Physiol.

[55] van Wijck K, Lenaerts K, van Loon LJ (2011). Exercise-induced splanchnic hypoperfusion results in gut dysfunction in healthy men. PLoS One.

[56] Fujita S, Dreyer HC, Drummond MJ (2009). Essential amino acid and carbohydrate ingestion before resistance exercise does not enhance postexercise muscle protein synthesis. J Appl Physiol.

[57] Beelen M, Burke LM, Gibala MJ (2011). Nutritional strategies to promote postexercise recovery. Int J Sport Nutr Exerc Metab.

[58] Wilkinson SB, Phillips SM, Atherton PJ (2008). Differential effects of resistance and endurance exercise in the fed state on signalling molecule phosphorylation and protein synthesis in human muscle. J Physiol.

[59] Beelen M, Tieland M, Gijsen AP (2008). Coingestion of carbohydrate and protein hydrolysate stimulates muscle protein synthesis during exercise in young men, with no further increase during subsequent overnight recovery. J Nutr.

[60] Res PT, Groen B, Pennings B (2012). Protein ingestion prior to sleep improves post-exercise overnight recovery. Med Sci Sports Exerc.

[61] Groen BB, Res PT, Pennings B (2011). Intragastric protein administration stimulates overnight muscle protein synthesis in elderly men. Am J Physiol.

[62] Ivy J, Res P, Sprague R (2003). Effect of a carbohydrate–protein supplement on endurance performance during exercise of varying intensity. Int J Sport Nutr Exerc Metab.

[63] Saunders M, Kane M, Todd M (2004). Effects of a carbohydrate–protein beverage on cycling endurance and muscle damage. Med Sci Sports Exerc.

[64] Saunders M, Luden N, Herrick J (2007). Consumption of an oral carbohydrate–protein gel improves cycling endurance and prevents postexercise muscle damage. J Strength Cond Res.

[65] Lee J, Maughan R, Shirreffs S (2008). Effects of milk ingestion on prolonged exercise capacity in young, healthy men. Nutrition.

[66] Martinez-Lagunas V, Ding Z, Bernard J (2010). Added protein maintains efficacy of a low-carbohydrate sports drink. J Strength Cond Res.

[67] Romano-Ely B, Tod M, Saunders M (2006). Effect of an isocaloric carbohydrate–protein–antioxidant drink on cycling performance. Med Sci Sports Exerc.

[68] Saunders M, Moore R, Kies A (2009). Carbohydrate and protein hydrolysate coingestions improvement of late-exercise time-trial performance. Int J Sports Nutr Exerc Metab.

[69] Valentine R, Saunders M, Todd M (2008). Influence of carbohydrate–protein beverage on cycling endurance and indices of muscle disruption. Int J Sport Nutr Exerc Metab.

[70] Currell K, Jeukendrup AE (2008). Validity, reliability and sensitivity of measures of sporting performance. Sports Med.

[71] Breen L, Tipton K, Jeukendrup A (2010). No effect of carbohydrate–protein on cycling performance and indices of recovery. Med Sci Sports Exerc.

[72] Madsen K, MacLean D, Kiens B (1996). Effects of glucose, glucose plus branched-chain amino acids, or placebo on bike performance over 100 km. J Appl Physiol.

[73] Osterberg K, Zachwieja J, Smith J (2008). Carbohydrate and carbohydrate + protein for cycling time-trial performance. J Sports Sci.

[74] van Essen M, Gibala MJ (2006). Failure of protein to improve time trial performance when added to a sports drink. Med Sci Sports Exerc.

[75] van Hall G, Raaymakers JSH, Saris WHM (1995). Ingestion of branched chain amino acids and tryptophan during sustained exercise in man: failure to affect performance. J Physiol.

